# Postural balance and functional mobility in relation to BMI and body composition among female students at a College of Applied Medical Sciences: A cross-sectional study

**DOI:** 10.1016/j.clinsp.2024.100401

**Published:** 2024-06-05

**Authors:** Muneera Mohammed Almurdi

**Affiliations:** Rehabilitation Health Sciences Department, College of Applied Medical Sciences, King Saud University, Saudi Arabia

**Keywords:** Postural balance, Functional mobility, BMI, Body Composition, Students

## Abstract

**Introduction:**

This study aimed to compare different Body Mass Index (BMI) categories, body composition parameters, postural balance, and functional mobility among female students at a College of Applied Medical Sciences; and examine the relationship between BMI and body composition with postural balance and functional mobility among female students at the college of applied medical sciences.

**Materials and methods:**

Female students, aged 18‒25 years old. They were subdivided into four groups according to their BMI category: underweight, normal, overweight, or obese, with *n* = 20 participants in each group. A Bioelectrical Impedance Human Body Analyzer (BIA) was used to assess the following body composition parameters: Fat Percentage (FATP), fat mass, muscle mass, and Total Body Water (TBW). A NeuroCom Balance Master was used to assess postural balance and functional mobility. A Timed Up and Go (TUG) test was employed to assess functional mobility. All data were analyzed using SPSS.

**Results:**

Participants’ ages and heights were matched (*p* > 0.05). However, there were variables that were statistically significantly higher in terms of weight, BMI and body composition, including FATP, fat mass, muscle mass, and TBW (*p* = 0.000), among the obese group. Moreover, postural balance was lower among the obese group. There was a significant relationship between BMI and body composition variables in respect of postural balance but not in relation to functional mobility.

**Conclusion:**

Postural instability but not functional mobility was related to higher BMI and body composition values among the study groups.

## Highlights

• Postural balance was reduced in higher BMI students.
• BMI was correlated with postural balance but not functional mobility.
• Body composition was correlated with postural balance but not functional mobility.
• Exploring the factors that contribute to the disturbance of postural balance and functional mobility is recommended.

## Introduction

Postural balance is an essential component for keeping the human body stable and in an upright and erect position. There are two types of postural balance: static and dynamic. Static balance keeps the human body stable at rest without movement, whereas dynamic balance allows movement.^1^ Balance can be defined as the procedure to keep the center of Gravity (COG) with the body's base of support provided by muscular function and joint position.^2^ Balance can be controlled by the integration of sensory and motor information from the visual, auditory and somatosensory systems to the central nervous system and muscle function is activated to maintain postural stability. Moving the body's COG out of the base of support can cause postural sway or instability.^3^

Functional mobility is a term describing the subject's ability to move and transfer from one location to another to achieve daily living activities. It encourages subjects to be physically active at home, school and in the community, which contributes to health and quality of life.^4^

The Body Mass Index (BMI) is defined as a person's body weight divided by the square of that person's height.^5^ BMI is categorized based on the World Health Organization (WHO) classification as follows: BMI <18.5 kg/m^2^ = Underweight; 18.5‒24.9 kg/m^2^ = Normal weight; 25‒27.9 kg/m^2^ = Overweight; and >28 kg/m^2^ = Obese.^6^ Increased BMI is related to postural instability in static balance with open and closed eyes^7^^,^^8^ and dynamic balance.^8^

The obesity ratio has grown for adults and adolescents to 27.5 % and 47.1 %, respectively, during the last 30 years, with currently over 400 million individuals suffering from obesity worldwide.^9^ Similarly, according to the WHO, it was estimated that more than 340 million children and youths aged 5‒19 years were overweight or obese around the world.^10^ In Saudi Arabia, around one-third of adults are obese, and obesity is one of the main health problems affecting all ages and genders.^11^

Women have less muscle mass compared to men, leading to a reduced ability to burn calories; accordingly, it is easier for women to have more fat and to gain weight.^12^ An increase in BMI in the overweight and obese criteria for university students has become a major challenge due to reduced physical activity and an unhealthy diet.^13^ An unhealthy lifestyle is trending among university students; thus, it is important to encourage institutions to apply healthy educational programs for college students.^14^

Psychological stress is an additional factor in increasing obesity at the university level particularly for medical students.^15^^,^^16^ A study by Makkawy et al. (2021) of 433 medical and dentistry students found there was a high prevalence of overweight (23.7 %) and obesity (11 %) among students at a health science college at Dar Al Uloom University in Saudi Arabia.^17^ This result was higher than that found in another study in Saudi Arabia among Northern Border University students, which found that 21.7 % were overweight and 8.4 % were obese.^18^ Moreover, a study in Iraq among Kerbala University students found that 22.9 % of the students were overweight and 5.6 % were obese.^19^ A study conducted at Ain Shams University in Egypt reported 13.4 % of their students were obese.^20^

Body composition is defined as the distribution of body mass between three separate compartments: fat-free tissue or lean body mass, extracellular water, and adipose tissue. Increased adipose tissue and body mass can reduce postural balance. Thus, good postural balance and body composition are key components of health in populations.^21^

It is reported that extra fat tissue, particularly in the abdominal area, leads to abnormal weight distribution in the body segments and incorrect biomechanics^22^ causing a disturbance in postural stability during walking.^23^ This is related to an increased possibility of falling, injuries, and disabilities.^24^ It has also been reported that increased BMI was associated with abnormal walking characteristics. Obese subjects have a slower walking speed, wider base of support, shorter step length, and greater step width in order to maintain balance during walking.^25^

Previous studies have demonstrated that balance, functional capacity and quality of life were improved in obese individuals after losing weight and engaging in a physical activity program. This confirms the importance of losing weight and being physically active in order to improve balance and prevent falls. Moreover, musculoskeletal injuries could be avoided and the need for medical care, especially for physical therapy services, and healthcare costs could be minimized.

Medical students need to be fit and physically active. Increased BMI among the population in Saudi Arabia with common health complications, which were mainly for postural stability but not for functional mobility, has been investigated in the scientific literature. In a recent literature review, varying results were found in different countries, such as Bangladesh,^25^ Indonesia,^1^ Korea,^22^ and India.^8^ Evidence suggests that individuals with higher BMI and body composition are more likely to experience postural balance problems based on different cultures, age groups, gender or used assessment methods.^8^

There are limited studies addressing increased BMI and body composition parameters and the relationship of postural balance and functional mobility among young Saudi female students at a health sciences college. Therefore, this study aimed to accomplish the following: 1) Compare different BMI categories, body composition parameters, postural balance, and functional mobility among female students at a college of applied medical sciences; and 2) Examine the relationship between BMI and body composition with postural balance and functional mobility among female students at the college of applied medical sciences.

This research can have significant implications for healthcare, rehabilitation, and fitness, among other fields. By understanding the factors that contribute to poor postural balance and decreased functional mobility, professionals can develop effective exercise programs tailored to each individual's needs. In addition to improving balance and overall fitness (physical activities), these programs can improve intervention outcomes and also reduce the risk of falls, prevent occurring injuries and promote health and well-being among individuals.

## Materials and methods

### Study design and setting

A cross-sectional study. The STROBE checklist was followed for reporting this study.

### Participants and sample size

Female students studying at the College of Applied Medical Sciences at King Saud University in Riyadh, Saudi Arabia, aged 18‒25 years old, with BMI >18 kg/m^2^ were recruited to assess their postural balance and functional mobility. Participants were subdivided into four groups according to their BMI category: underweight, normal weight, overweight, or obese, with *n* = 20 participants in each group. Students with neurological problems, lower limb injuries or who had undergone surgery in the previous 6 months, knee or ankle clinical instability, arthritis, or suffering from rheumatic or vestibular disease, visual or hearing impairments and pregnant students were excluded from participation in this study.

### Sample size

A convenience sampling method was used. The sample size was calculated using online calculator.net (www.calculator.net/sample-size-calculator.html). It was found that 80 students needed to be included in the study, with a confidence interval of 95 % and a margin of error of 5 %. The sample size was calculated based on previous studies.^25^

### Ethical considerations

The Institutional Review Board approved the study in the College of Medicine at King Saud University (Ref. n° 21/0924/IRB). Prior to running the study measurements, the study aims, and procedure were explained verbally by the researcher and the written consent form was read and signed by the participants. The participants’ information was kept private, and none of their names or other identifying information was disclosed.

### Instruments

Height and weight were measured using a Detecto 750 Scale (E01614–0194) to calculate BMI. The following body composition parameters were measured for each of the participants, using a Bioelectrical Impedance Human Body Analyzer (Tanita Body Composition Analyzer BC-418) (Tanita, Tokyo, Japan): Fat Percentage (FATP), fat mass, muscle mass (Fat-Free Mass [FFM]) and Total Body Water (TBW). Participants’ postural balance and functional mobility were assessed using a NeuroCom Balance Master (version 8.2.0). Postural sway in static standing balance was examined using the modified Clinical Test of Sensory Interaction in Balance (mCTSIB), a test designed to detect sensory deficiency during standing. Dynamic standing balance was assessed by a Limit of Stability (LOS) test, to detect motor dysfunction in a standing position. The LOS assessment included three tests: Reaction Time (RT), Movement Velocity (MV) and Directional Control (DC). Functional mobility was evaluated by Step Up and Over (SUO) and Sit-To-Stand (STS) tests.^26^ These are reliable and valid tools that allow clinicians to assess the sensory, automatic, and voluntary motor components of balance control.^27^ In addition to the Balance Master instrument, a Timed Up and Go (TUG) test was used to assess functional mobility and dynamic balance during walking. This is a reliable, economical, safe, and time-efficient outcome measure.^28^

### Procedure

After receiving ethical approval for this study, an advertising banner with the researcher's contact information was distributed in the main hall of the college. Students contacted the researcher by phone and/or email to ascertain their eligibility to be included in the study based on the inclusion criteria. Following this stage, the researcher booked an appropriate appointment for one session with each student separately.

All the study measurements were conducted in one session and in the same room in the Physical Therapy Department laboratory at the college. Participants’ demographic data were recorded. All participants were instructed to remove their shoes before undertaking the study tests to prevent the effect of shoes and to make sure the measurements were accurate. Height and weight were measured, and BMI was calculated to classify the present study groups based on their BMI. All participants started with a body composition assessment followed by Balance Master measurements, beginning with a static balance test, followed by a dynamic test, and then completed functional mobility tests. The session ended with a TUG assessment. For the Balance Master and TUG assessments, one training trial for each test was permitted before data recording.

### Body composition assessment

Participants were asked to stand on the Bioelectrical Impedance Human Body Analyzer with bare feet and with the soles of their feet placed correctly on the electrodes of the machine platform. They were asked to grasp the handle electrodes firmly with their hands and fully extended elbows and slightly abducted shoulders of around 30° and to maintain this stable position for approximately 1 min.^29^ BMI, FATP, fat mass, FFM, and TBW were analyzed and the results were printed out automatically from the device.

### Balance master assessment

#### Postural balance

##### Modified clinical test of sensory interaction in balance (mCTSIB) (static balance)

The mCTSIB was used to examine static standing balance through assessment of the sway velocity of the center of Pressure (COP) for four sensory tests that grew gradually more difficult, each test containing three trials lasting 10 s, for each test one score was calculated by averaging these three trials. The four tests were as follows: 1) Standing upright with eyes open on a firm surface; 2) Standing with eyes closed on a firm surface; 3) Standing with eyes open on a foam surface; and 4) Standing with eyes closed on a foam surface. The test order was the same for all participants. The participants were recommended to stand as upright and stable as they could, putting their arms by the sides of their bodies. During the eyes open test trials, the participants were asked to keep their eyes open and to look forward and in an erect position. During the closed eyes test, they were blindfolded while standing upright and asked to keep as stable as possible with closed eyes. The sway velocity of the COG was indicated for static stability, with a higher postural sway presenting static balance instability and vice versa.^26^

##### Limit of stability (LOS) (dynamic balance)

The LOS test depends on voluntary trunk movement in a standing position in a number of directions following a target and maintaining a stable position for a short period of time. This test measures participants’ ability to control their COG within the base of support. It evaluates the maximum distance which participants can lean voluntarily in a certain direction without losing stability. Starting from the center on the screen eight targets were required to reach as possible (forward, forward-right, right, backward-right, backward, backward-left, left, and forward left). Participants were asked to move as quickly and correctly as possible in these directions without flexing the trunk or moving the arms and feet. The mean value of these eight targets was recorded.

### Functional mobility assessment

#### Step Up and Over (SUO) test

The SUO test evaluates participants’ ability to step up onto a curb with one foot, raising the body in an upright position, and swinging the other foot over the curb and lowering the body on the force plate. Three trials were achieved and the mean value of these three trials was recorded as one score.

#### Sit-To-Stand (STS) test

The STS test measures participants’ ability to stand up from a sitting position on a bench, relaxing their upper limbs at their sides. The movement should be done as quickly as possible without using their upper extremities or any other assistive devices. Three repeated trials were performed and the mean of these three trials was recorded as one score.

#### Timed Up and Go (TUG) test

The TUG test was used to determine functional mobility and dynamic balance while walking and is a reliable method (intraclass correlation coefficient = 0.96‒0.98).^30^ This test was performed as quickly and safely as possible. An adhesive tape was used as a marker on the floor with 3 m distance from the used chair for this test. The participants were instructed to sit in an armchair with their back against the chair, then asked to rise from the chair and walk for around 3 m, and then turn around and walk back to the chair and sit down, the walking time was recorded using a stopwatch (one trial was recorded).^8^^,^^28^

### Data processing

Data collection was undertaken over a period of 4 months, from October 2021 to January 2022.

### Statistical analysis

Statistical analyses were performed using IBM SPSS Statistics for Windows v28.0 (IBM Corp., Armonk, NY). The Shapiro-Wilk test was conducted to assess the normality of the data distributions. Data are presented as the mean ± Standard Deviation (SD) for normally distributed data or the median for skewed data. Based on the Shapiro-Wilk test, an analysis of variance (ANOVA) Kruskal-Wallis non-parametric test was performed to assess the differences in the measured variables among the four study groups. The correlation test was ascertained using the non-parametric Spearman correlation test. The correlation coefficients were interpreted as follows: *r* < 0.1: no correlation; *r* = 0.1‒0.3: weak correlation; *r* = 0.4‒0.6: moderate correlation; and *r* = 0.7‒0.8: strong correlation.^31^

## Results

A total of 80 adult female students were recruited for this study. They were subdivided into four groups based on their BMI category: underweight, normal weight, overweight, and obese, with 20 participants in each group. Participants’ age (20.18 ± 1.14 years old) and height (158.76 ± 5.27 cm) were matched (*p* > 0.05) among all the study groups. However, there were significant differences in their weight (kg) (63.89 ± 18.78), BMI (kg/m^2^) (25.33 ± 7.35) and body composition variables, including FATP (32.51 ± 11.43), fat mass (kg) (22.82 ± 13.97), FFM muscle mass (kg) (41.12 ± 5.28), and TBW (kg) (34.52 ± 2.78) (*p* = 0.000), as shown in Table 1. Those in the obese group had significantly higher values for weight, BMI, and body composition variables.

**Table 1:** Participants’ demographic characteristics, BMI and body composition among 80 female students.

Group Variable	Underweight	Normal weight	Overweight	Obese	Total	p-value
	(*n* = 20) mean ± SD	(*n* = 20) mean ± SD	(*n* = 20) mean ± SD	(*n* = 20) mean ± SD	(*n* = 80) mean ± SD	
Age (years)	20.30 ± 1.12	20.30 ± 0.92	19.85 ± 0.98	20.30 ± 1.49	20.18 ± 1.14	0.452
Height (cm)	157.65 ± 6.17	160.60 ± 5.21	158.20 ± 4.70	158.60 ± 4.79	158.76 ± 5.27	0.32
Weight (kg)	42.97 ± 3.87	55.77 ± 6.87	67.54 ± 5.33	89.29 ± 12.36	63.89 ± 18.78	0
BMI (kg/m²)	17.44 ± 0.98	21.49 ± 1.75	26.96 ± 1.38	35.43 ± 5.26	25.33 ± 7.35	0
Body composition						
FATP	18.40 ± 4.42	27.91 ± 5.41	36.92 ± 3.67	46.83 ± 3.63	32.51 ± 11.43	0
Fat mass (kg)	8.07 ± 2.48	15.86 ± 4.64	25.10 ± 4.12	42.26 ± 9.13	22.82 ± 13.97	0
Muscle mass (kg) (FFM)	35.03 ± 2.19	39.92 ± 3.16	42.37 ± 2.49	47.16 ± 3.79	41.12 ± 5.28	0
TBW	25.65 ± 1.61	29.23 ± 2.31	31.13 ± 1.62	34.52 ± 2.78	34.52 ± 2.78	0

BMI, Body Mass Index; FATP, Fat Percentage; FFM, Fat Free Mass; TBW, Total Body Water. An analysis of variance (ANOVA) Kruskal-Wallis test was used to assess the differences in the measured variables among the four study groups. The significance level set at *p* < 0.05.

A description and the statistical differences in the postural sway and functional mobility variables among all study groups are presented in Table 2. The table shows that there was a statistically significant difference in static balance (postural sway), particularly with the sway foam surface test, in both conditions with open and closed eyes (*p* = 0.006 and *p* = 0.005, respectively). Moreover, there was significantly lower DC in the LOS test (dynamic balance) in the obese group, whereas there was no significant difference in the other LOS variables (RT and MV). In terms of functional mobility variables, there were no statistically significant differences between groups in terms of the SUO, STS and TUG variables, apart from the Step Up Onto Rising (SUOR) time: there was a significant reduction in SUOR time in the overweight group (*p* = 0.041), and STS velocity (*p* = 0.016), there was a significantly longer time in terms of STS velocity in the overweight group, as shown in Table 2.

**Table 2:** Differences in postural balance (static and dynamic balance) and functional mobility among the study groups.

Group Variable	Underweight (*n* = 20) mean ± SD	Normal weight (*n* = 20) mean ± SD	Overweight (*n* = 20) mean ± SD	Obese (*n* = 20) mean ± SD	Total (*n* = 80) mean ± SD	p-value
**Postural balance (static and dynamic) balance Static balance**						
Sway firm EO	0.36 ± 0.20	0.31 ± 0.13	0.33 ± 0.15	0.35 ± 0.17	0.34 ± 0.16	0.82
Sway firm EC	0.34 ± 0.21	0.29 ± 0.11	0.28 ± 0.09	0.30 ± 0.09	0.30 ± 0.14	0.681
Sway foam EO	0.57 ± 0.12	0.45 ± 0.09	0.47 ± 0.18	0.49 ± 0.17	0.49 ± 0.15	0.006
Sway foam EC	0.75 ± 0.21	0.68 ± 0.12	0.56 ± 0.15	0.57 ± 0.16	0.64 ± 0.18	0.005
**Limit of stability (LOS) tests (dynamic balance)**						
RT	1.12 ± 0.35	0.94 ± 0.39	1.01 ± 0.36	1.15 ± 0.30	1.05 ± 0.35	0.121
MV	3.82 ± 1.22	3.90 ± 1.01	4.10 ± 1.38	3.61 ± 1.03	3.86 ± 1.16456	0.611
DC	84.25 ± 12.95	84.10 ± 21.42	79.37 ± 8.46	75.90 ± 7.23	80.90 ± 13.89	0
**Functional mobility tests**						
SUOL index	23.17 ± 5.59	25.15 ± 7.35	27.61 ± 6.69	25.46 ± 6.94	25.34 ± 6.739	0.31
SUOL time (*sec*)	1.91 ± 0.53	1.80 ± 0.40	1.62 ± 0.32	1.81 ± 0.48	1.79 ± 0.44	0.279
SUOL impact index	32.10 ± 14.84	30.11 ± 11.03	32.57 ± 9.89	24.61 ± 9.07	29.85 ± 11.65	0.108
SUOR index	29.26 ± 9.94	31.21 ± 10.06	31.88 ± 9.15	30.04 ± 9.22	30.60 ± 9.47	0.849
SUOR time (*sec*)	1.64 ± 0.31	1.62 ± 0.34	1.44 ± 0.20	1.60 ± 0.53	1.57 ± 0.37	0.041
SUOR impact index	32.86 ± 12.54	32.55 ± 12.59	33.28 ± 11.55	26.61 ± 9.30	31.32 ± 11.68	0.244
STS transfer time (*sec*)	0.67 ± 0.32	0.67 ± 0.54	0.43 ± 0.20	0.61 ± 0.45	0.60 ± 0.40	0.175
STSBW%, rising index	13.91 ± 5.32	14.90 ± 5.52	13.69 ± 5.21	12.38 ± 4.44	13.72 ± 5.12	0.5
STS, velocity, °/s	1.99 ± 0.85	2.39 ± 1.01	3.06 ± 1.02	2.75 ± 1.38	2.55 ± 1.14	0.016
TUG	8.99 ± 1.47	9.08 ± 1.24	9.03 ± 1.26	9.70 ± 2.61	9.20 ± 1.73	0.561

EO, Eyes Open; EC, Eyes Closed; LOS, Limit of Stability; RT, Reaction Time; MV, Movement Velocity; DC, Directional Control; SUOL, Step Up Onto Lowering; SUOR, Step Up Onto Rising; STS, Sit To Stand; STSBW, Sit to Stand Body Weight; TUG, Timed Up and Go; *sec*, seconds.

An analysis of variance (ANOVA) Kruskal-Wallis test was used to assess the differences in the measured variables among the four study groups. The significance level set at *p* < 0.05.

### Relationship of BMI and postural balance with functional mobility

The relationship between BMI, postural balance and functional mobility was assessed among all participants. There was a significant negative moderate correlation between BMI and postural sway in the sway foam surface test in both conditions of eyes open and closed (*rs* = −0.33, *p* = 0.003 and *rs* = −0.42, *p* = 0.000, respectively). With regard to the LOS test, there was no significant correlation between BMI and RT and MV, although there was a significant negative moderate correlation between BMI and DC (*rs* = −0.43, *p* = 0.000) among all participants. No statistically significant correlation was found between BMI and the functional mobility variables, apart from STS velocity (*rs* = 0.33, *p* = 0.03).

The relationship of BMI with postural balance and functional mobility between study groups based on their BMI classification was analyzed and is shown in Table 3. It can be seen that there was a significant negative strong-to-moderate correlation between BMI and postural balance in the sway foam surface test with eyes open and closed (*rs* = −0.73, *p* = 0.000 and *rs* = −0.43, *p* = 0.056, respectively) and only for eyes open in the sway firm surface test (*rs* = −0.47, *p* = 0.035) among the obese group. There was a significant negative correlation (*rs* = −0.45, *p* = 0.046) in the sway firm surface test with eyes closed in the overweight group. The SUOL and SOUR times and STS velocity were also significantly correlated (*p* < 0.05) among the normal weight group. However, there was no statistically significant correlation in the other variables measured among the study groups, as shown in Table 3.

**Table 3:** BMI in relation to postural balance (static, postural sway) and dynamic balance (LOS tests) and functional mobility (SUO, STS and TUG) between study groups.

BMI	Underweight [*rs*, p]	Normal weight [*rs*, p]	Overweight [*rs*, p]	Obese [*rs*, p]
Sway firm EO	0.30, 0.207	−0.04, 0.871	−0.24, 0.313	−0.47, 0.035
Sway firm EC	−0.09, 0.702	−0.12, 0.609	−0.45, 0.046	−0.36, 0.119
Sway foam EO	0.06, 0.803	−0.03, 0.911	−0.32, 0.172	−0.73, 0.000
Sway foam EC	−0,11, 0.633	−0,28, 0.227	−0.19, 0.422	−0.43, 0.056
LOSRT	0.08, 0.726	0.03, 0.907	0.25, 0.287	−0.32, 0.174
MV	0.25, 0.287	0.16, 0.506	0.11, 0.648	0.24, 0.311
DC	−0.21, 0.364	0.11, 0.643	−0.04, 0.864	0.22, 0.354
SUOL index	0.03, 0.897	0.27, 0.245	−0.02, 0.940	−0.24, 0.316
SUOL time (*sec*)	0.09, 0.700	−0.44, 0.053	−0.03, 0.917	0.07, 0.816
SUOL impact index	0.05, 0.835	−0.15, 0.518	0.28, 0.238	−0.30, 0.192
SUOR index	−0.35, 0.133	0.14, 0.510	−0.15, 0.520	0.22, 0.342
SUOR time (*sec*)	0.03, 0.900	−0.45, 0.045	0.22, 0.348	0.07, 0.765
SUOR impact index	−0.21, 0.376	0.21, 0.387	0.14, 0.551	0.03, 0.916
STS transfer	−0.06, 0.803	−0.53, 0.017	0.20, 0.387	0.03, 0.892
STSBW	−0.42, 0.064	0.21, 0.387	−0.19, 0.422	−0.17, 0.485
STS velocity	0.28, 0.240	0.48, 0.031	0.19, 0.411	0.06, 0.810
TUG	−0.11, 0.636	−0.39, 0.086	−0.24, 0.306	0.35, 0.128

BMI, Body Mass Index; EO, Eyes Open; EC, Eyes Closed; LOS, Limit of Stability; RT, Reaction Time; MV, Movement Velocity; DC, Directional Control; SUOL, Step Up Onto Lowering; SUOR, Step Up Onto Rising; STS, Sit To Stand; STSBW, Sit To Stand Body Weight; TUG, Timed Up and Go.

Spearman correlation test (*rs*) was used, the significance level was set at *p* < 0.05.

### Body composition variables in relation to postural balance and functional mobility among all study groups

Body composition variables, which included FATP, fat mass, FFM and TWB, were analyzed in relation to postural balance and functional mobility in all participants. It was found that FATP, fat mass, FFM and TBW were significantly negatively correlated with the results of the sway foam test with eyes open and closed, STS velocity, DC (*p* < 0.01) and STS transfer (*p* < 0.05). However, there was no statistically significant correlation with the other measured variables (*p* > 0.05).

### Body composition variables in relation to postural balance and functional mobility between study groups (underweight, normal weight, overweight, and obese)

Among those in the underweight group, FATP, fat mass and TWB were significantly correlated with the following functional mobility variables: SUO index (*p* < 0.01), STSBW and STS velocity (*p* < 0.05). For those in the normal weight group, FATP and fat mass were significantly correlated with SUOL index, SUOR time, SUO impact index, STS transfer and STS velocity (*p* < 0.05). However, there was no significant correlation with the other measured variables. Furthermore, there was no significant correlation between FFM and TBW and any of the postural balance and functional mobility variables (*p* > 0.05).

With regard to the overweight group, FATP and fat mass were significantly negatively correlated with the results of the sway firm surface test with eyes closed (*rs* = −0.487, *p* = 0.029 and *rs* = −0.45, *p* = 0.04, respectively). FFM and TBW were significantly correlated with the SUOR impact index (*rs* = 0.48, *p* = 0.031 and *rs* = 0.48, *p* = 0.030, respectively). There was no significant correlation with the other variables measured. In the obese group, FATP and fat mass were significantly correlated with the results for postural sway in both tests on a firm surface (*p* < 0.05) and the sway foam surface tests with eyes open and closed (*p* < 0.01). FFM and TBW were significantly negatively correlated with the results of the sway firm surface test with eyes closed and the sway foam surface test with eyes open (*p* < 0.05) and significantly negatively correlated with MV (*p* < 0.05), whereas there was no significant correlation with the other variables measured.

## Discussion

This cross-sectional study compared differences in BMI category, body composition parameters, postural control, and function mobility among female students at the College of Applied Medical Sciences. The study found that there were statistically significant greater differences in weight, BMI, and body composition variables (FATP, fat mass, FFM and TBW) in the obese group. There were significant differences in static and dynamic balance with eyes open and closed, especially for the sway foam surface test and in the LOS test (dynamic balance) in terms of DC, but not in the others (RT and MV) for LOS in the overweight and obese groups. However, there were no significant differences in the results of the functional mobility test whether using the Balance Master SUO, STS and TUG tests between study groups.

There was a significant negative correlation between BMI and postural balance. However, there was no significant correlation between BMI and functional mobility tests and TUG test results.

### BMI and body composition among the study groups

This study found that the obese group had a higher BMI, which is in line with another study conducted in Riyadh among health science college students.^17^ This was explained by changes in eating habits to unhealthy food and reduced physical activity.^17^ It was also found among King Saud University students (*n* = 312) that the majority of their calorific consumption (72 %) was in the form of carbohydrates and there was a high intake of meat for protein.^32^ Other studies confirm a high consumption of unhealthy food, such as fried and fast foods among adults in Saudi Arabia.^33^ One study found that 57 % of the participants consumed fast food weekly and 43 % drank soft drinks more than once a day.^33^ Another possible reason for the study participants increasing their BMI is reduced physical activities, spending more time indoors sitting using their computers or spending time using social media on their smartphones, which is in line with other studies.^17^^,^^34^ Moreover, psychological stress during the academic university year was an important factor in increasing BMI among university students.^15^^,^^17^

FATP, fat mass, FFM, and TBW were significantly higher in the obese group in this study. This is in agreement with another study that found that obese students had a significantly higher water intake: they had a significantly greater number of cups of water per day compared to those in a non-obese group.^17^

### Postural balance and functional mobility parameters

This study found there was significant postural sway (postural disability) in static balance, especially in more challenging conditions when using a sway foam surface for both the eyes open and closed states, and in terms of dynamic balance in the obese group, which was similar to other findings.^8^^,^^23^^,^^35^ These results for postural instability were revealed despite the intact nature of sensory and motor information from the visual, auditory and somatosensory systems. Therefore, one possible reason for this is increased BMI. This would mean abnormal weight distribution in the body segments, particularly in the abdominal area, where fat tissue accumulates, causing increased anterior pelvic tilt with increased lumber extension, leading to abnormal biomechanics in the muscles and joints. This encourages the COG to move abnormally forward to the front of the feet in order to keep the person stable; thus, postural instability could occur. This possible cause is supported by other studies.^25^^,^^36^

### BMI and body composition variables in relation to postural balance and functional mobility among all study groups

This study found a significant negative moderate-to-strong correlation between BMI and postural balance only for static balance with eyes open and closed (*p* < 0.05) using sway firm and sway foam surfaces among the overweight and obese groups. This is in line with another study by Handayani et al. (2022) among 49 Indonesian students at the Sports Martial Arts Malikussaleh University. The researchers found that BMI was related to postural balance (static balance) among the students.^1^ On the other hand, there was no significant correlation between BMI and dynamic balance and functional mobility measure variables.

Body composition parameters were significantly negatively correlated with static and dynamic balance but not for the functional mobility variables among the overweight and obese groups, which is in line with a previous study.^1^ It has also been confirmed that the accumulation of excess adipose tissue in the body causes muscle weakness and atrophy, leading to a disturbance in postural stability.^37^ Fat tissue accumulation stimulates muscle protein deprivation, causing muscle atrophy and reduced muscle protein synthesis, leading to lower muscle strength^38^ and functional mobility.^39^ Thus, postural disability could occur as a result of impairment in muscle function due to excess fat tissue deposition within the muscles among obese subjects.^40^ However, the results of the present study were contrary to other research, which found no correlation between fat mass and postural balance in either open or closed eyes conditions among adult females.^35^ A possible explanation for these conflicting results was that the female participants’ BMI (23.2 kg/m^2^) was lower in the other study than in the overweight and obese groups’ BMI categories (35.43±5.26 kg/m^2^, 26.96 ± 1.38 kg/m^2^, respectively).

### Study limitations

This study has a number of limitations. The study involved a small sample size and was restricted to young female participants, and thus the results will be valid only among this population. Including male participants would have allowed the study to assess statistical differences in terms of gender. A larger sample size is also needed.

## Conclusion

This study found that increased BMI and the deposition of unnecessary fat mass within muscular tissue were significantly related to postural balance for both static and dynamic balance with eyes open and closed, but not functional mobility among female students. Future research is needed to explore related factors that might affect postural balance and functional mobility among the population.

## Author's contributions

MMA contributed to the conceptualization of the study, methodology, data analysis, investigation, data coding, writing the original draft and revising and editing the manuscript. MMA read and agreed to the published version of the manuscript.

## Funding

This research did not receive any specific grant from funding agencies in the public, commercial, or not-for-profit sectors.

## Declaration of competing interest

The authors declare no conflicts of interest.
